# The syndrome of progressive posterior cortical dysfunction: A multiple case study and review

**DOI:** 10.1590/S1980-57642008DN10300014

**Published:** 2007

**Authors:** Renata Areza-Fegyveres, Paulo Caramelli, Claudia Sellitto Porto, Carla Rachel Ono, Carlos Alberto Buchpiguel, Ricardo Nitrini

**Affiliations:** 1 Neurologist, member of the Behavioral and Cognitive Neurology Unit, Department of Neurology, University of São Paulo School of Medicine, São Paulo, SP, Brazil.; 2 Associate Professor of Neurology, Department of Internal Medicine, Faculty of Medicine, Federal University of Minas Gerais, Belo Horizonte, MG, Brazil.; 3 Neuropsychologist, member of the Behavioral and Cognitive Neurology Unit of the University of São Paulo School of Medicine.; 4 Nuclear Medicine Physician, Division of Nuclear Medicine, Department of Radiology, University of São Paulo School of Medicine.; 5 Associate Professor, Department of Radiology (Nuclear Medicine), University of São Paulo School of Medicine.; 6 Associate Professor, Department of Neurology, University of São Paulo School of Medicine.

**Keywords:** posterior cortical atrophy, syndrome of progressive posterior cortical dysfunction, Alzheimer disease, visual symptoms

## Abstract

Dementia presenting with prominent higher order visual symptoms may be observed in a range of neurodegenerative conditions and is often challenging to diagnose.

**Objectives:**

To describe cases of progressive dementia presenting with prominent visual cortical symptoms.

**Methods:**

We conducted a retrospective search of cases of progressive dementia with predominant visual symptoms, seen at our dementia unit from 1996 to 2006.

**Results:**

Twelve patients (5 men, 7 women) were identified, with ages ranging from 49 to 67 years. At the first examination, the duration of the symptoms ranged from one to ten years and the Mini-Mental State Examination scores from 7 to 27. Eleven patients presented with predominant visuospatial symptoms (partial or complete Balint syndrome) and one with visuoperceptive impairment. Other reported manifestations were: constructional apraxia in 11 patients, partial or complete Gerstmann syndrome in ten, ideomotor apraxia in nine, hemineglect or extinction in four patients, alien hand phenomenon in three, and prosopagnosia in one patient. Memory loss was reported by ten patients, but was not the main complaint in any of these cases. Insight was relatively preserved in five patients even after a long period following the onset of symptoms. Six patients developed parkinsonism during evolution. Clinical diagnoses were possible or probable AD in seven patients, cortico-basal degeneration in four, and dementia with Lewy body in one.

**Conclusions:**

Clinicians should consider this condition especially in presenile patients with slowly progressive higher-order visual symptoms. Although described in association with different conditions, it may also occur in Alzheimer disease.

## Abstract (pt)

As demências que se apresentam predominantemente com sintomas visuais associativos podem ser observadas em diferentes condições neurodegenerativas, sendo seu diagnóstico muitas vezes desafiador.

**Objetivos:**

Descrever as principais características clínicas de pacientes com demência progressiva que se apresentam com sintomas visuais proeminentes.

**Métodos:**

Conduzimos estudo retrospectivo de casos com demência progressiva com sintomas visuais predominantes vistos no nosso ambulatório de demência, no período de 1996 até 2006.

**Resultados:**

Doze pacientes (5 homens e 7 mulheres) foram identificados, com idades variando entre 49 e 67 anos. Na primeira consulta, duração de sintomas variou de um a dez anos e a pontuação do Mini-Exame do Estado Mental variou de 7 a 27 pontos. Onze pacientes apresentaram sintomas visuoespaciais predominantes (Síndrome de Bálint parcial ou completa) e um apresentou alteração visuoperceptiva. Outras manifestações relatadas foram: apraxia construtiva em 11 pacientes, síndrome de Gerstmann parcial ou completa em 10 pacientes, apraxia ideomotora em nove, heminegligência ou extinção em 4 pacientes, fenômeno da mão alienígena em 3 e prosopagnosia em um paciente. Queixa de perda de memória foi referida em 10 pacientes, mas em nenhum deles como queixa principal. Juízo crítico era preservado em cinco pacientes até estágios moderados da doença. Seis pacientes desenvolveram parkinsonismo ao longo da evolução. O diagnóstico clínico foi DA possível ou provável em sete pacientes, degeneração corticobasal em quatro e demência com corpos de Lewy em um paciente.

**Conclusões:**

Os clínicos devem ter em mente essa condição especialmente em pacientes pré-senis com queixas visuais complexas e lentamente progressivas. Apesar de descritas em diferentes condições, pode ocorrer na doença de Alzheimer.

The past three decades have seen a body of reports on patients suffering neurodegenerative disease with disruption of visual processing together with relative preservation of memory and insight. In 1988, Benson et al. described five patients with predominant higher order visual signs and symptoms which they denominated posterior cortical atrophy (PCA).^1^ Their patients developed alexia, agraphia, visual agnosia, partial or complete Bálint and Gerstmann syndromes, as well as transcortical sensory aphasia. Memory, insight and judgment were relatively preserved up until moderate stages of the disease. No pathologic specimens were available for their study. The authors speculated whether the underlying pathologic condition included a variant of Alzheimer disease (AD), a lobar atrophy analogous to Pick’s disease or a new unrecognized entity.^1^

PCA comprises disorders of either visuospatial processing or visual recognition, resulting from the dysfunction of parieto-occipital and temporo-occipital pathways, respectively.^2^ Visuospatial processing disorders are more frequent, although both may occur in the same patient.

After Benson et al.^1^ characterized and named PCA as a distinct phenotype, there have been two other attempts to improve clinical diagnostic criteria based on the growing literature on this issue.^3,4^ Tang-Wai et al.^3^ proposed a subset of diagnostic criteria for PCA based on their neuropathological and clinical findings. In addition, they proposed to rename the syndrome based on its concept and not on imaging findings: the syndrome of posterior progressive cortical dysfunction (SPPCD).

While the clinical presentation is relatively homogeneous, multiple etiologies can account for this syndrome. Recent studies involving autopsied series of cases have shown that AD with atypical distribution of pathological lesions, corticobasal degeneration (CBD), Creutzfeldt-Jakob disease and dementia with Lewy bodies (DLB) might underlie this syndrome. The most common etiology by far is AD.^3,5-10^

The aim of the present study was to describe cases of progressive dementia that presented with prominent visual cortical symptoms in an outpatient clinic specialized in dementia.

## Methods

A retrospective search of the case files from 1996 to 2006 of the Behavioral and Cognitive Neurology Unit, which is linked to a public university hospital, was undertaken. Approximately 700 files were examined. Other possible cases seen by the members of the Unit at other sites, mainly their private offices, were also included in the investigation.

For the diagnosis of progressive posterior cortical dysfunction, the criteria proposed by Tang-Wai et al. were used.^3^ Briefly, patients had to present visual complaints in the absence of significant primary ocular disease, explaining symptoms with insidious onset and gradual progression, relative preservation of anterograde memory and insight early in the disorder, disabling visual impairment throughout the disorder, absence of stroke or tumor and any of the following findings: simultanagnosia with or without optic ataxia or ocular apraxia, constructional dyspraxia, visual field defect, environmental disorientation or any of elements of Gerstmann syndrome.

The neuroimaging data were collected from the patients’ charts retrospectively, without further review.

Diagnoses were established in a consensus meeting following established criteria for AD,^11^ CBD^12^ and DLB.^13^

## Results

Twelve patients, 5 men and 7 women, were identified. The main demographic features as well as the duration of the symptoms up prior to the first consultation and initial Mini-Mental State Examination (MMSE)^14,15^ scores are shown in Table 1. Eleven patients came from the Cognitive and Behavioral out-patient clinic of the university hospital and one from the private clinic of one of the authors.

**Table 1:** Demographic features of twelve patients with progressive dementia and prominent visual symptoms.

	Mean (SD*)	Median	Minimum/maximal values
Age at first consultation	59.42 (5.71)	60	49-67
Age at onset	55.27 (6.42)	53	48-65
Schooling years	8.36 (5.55)	4	3-16
Interval between onset of symptoms and first consultation (months)	52.73 (40.91)	36	12-120
Mini-Mental State Exam	14 (7.10)	12	7-27

* SD, standard deviation.

Complaints of visual impairment led five patients to seek an ophthalmologic consultation, before a neurological examination. Caregivers’ main visual complaints included: “she looks but doesn’t see”, “she doesn’t see objects in front of her and she keeps searching for them with her hands”, “he bumps into furniture and behaves like a blind person”, “he forgot one side of the body”.

**Figure 1:**
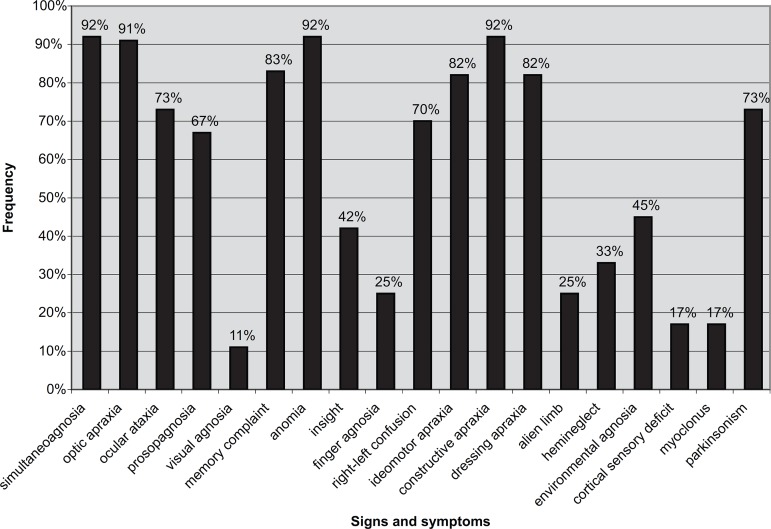
Frequency of signs and symptoms of twelve patients with SPPCD. *SPPCD, syndrome of progressive posterior cortical dysfunction.

All patients presented presenile onset (Table 1). The frequency of symptoms in our case series ranged according to the stage of the disease (Figure 1). Insight for the deficits was preserved in five patients. Complaints of memory were present in all of cases, but were not considered the main symptom. Nine patients presented partial Bálint syndrome at onset while two presented the full syndrome. At onset, three patients presented with partial Gerstmann syndrome. Hemineglect was present in four patients and appeared at late stages of disease. Ideomotor apraxia was present in nine subjects ranging from mild to severe. Eleven patients had constructional apraxia and nine patients had difficulty dressing.

Visual associative agnosia was reported in one patient. The most common behavioral and neuropsychiatric symptom was depressed mood which was reported by seven patients at the first outpatient visit. Parkinsonism was present in six patients: in five it was asymmetric and presented at early stages of the disease.

Educational level was very heterogeneous and ranged from 3 to 20 years. MMSE scores ranged from 7 to 27, most common errors being related to attention/calculation (12 patients), recall (12 patients), written command (9 patients) and drawing (12 patients). According to MMSE Brazilian normative data, 11 patients presented scores below those expected for their age and schooling.^15^ Only six out of 12 patients were able to complete the test of delayed recall of objects presented as line drawings from the Brief Cognitive Screening Battery,^16-18^ where severe visual perception impairment presented by the remaining six subjects hampered completion. Five patients showed low scores for delayed recall after the interference tasks. Category fluency (animals per minute)^19,20^ and Digit Span (forward and backwards)^21-23^ were found to be impaired in the nine patients.

Only five patients underwent complete neuropsychological evaluation resulting from the severity of the disease at first consultation.

All patients selected underwent conventional neurological and brief cognitive evaluations, neuroimaging and routine laboratory investigation. Structural imaging findings are reported in Table 2. Eleven patients had been submitted to magnetic resonance imaging (MRI) and one to computed cranial tomography (CCT). All patients presented varying degrees of diffuse cortical atrophy, ranging from mild to severe. Three patients also showed areas of focal atrophy (one occipital, one parietal and one frontotemporal).

**Table 2:** Structural and functional imaging findings of twelve patients with SPPCD*.

Case	Clinical diagnosis	Structural imaging (CCT^†^ or ^‡^MRI)	Functional imaging (conventional SPECT^§^)
1	^||^AD-SPPCD	^‡^MRI: diffuse severe atrophy	Temporo-parietal posterior hypoperfusion with extension to frontal regions bilaterally
2	^||^AD-SPPCD	^‡^MRI: diffuse moderate atrophy. Temporal lobes relatively preserved in contrast to hippocampal volume.	Not done
3	^||^AD-SPPCD	^‡^MRI: diffuse severe atrophy	Left occipital hypoperfusion
4	^||^AD-SPPCD	^‡^MRI: diffuse moderate atrophy	Hypoperfusion in both temporal lobes
5	^||^AD-SPPCD	^‡^MRI: mild leukoaraiosis and cortical atrophy especially in posterior lobes	Marked hypoperfusion in parietal lobes, especially to right with right fronto-temporal extension
6	^¶^CBD-SPPCD	^‡^MRI: diffuse atrophy, more marked in fronto-temporal regions	Diffuse cortical irregularity; hypoperfusion in high frontal regions
7	^¶^CBD-SPPCD	^‡^MRI: left parietal atrophy	Left temporo-parietal hypoperfusion
8	^¶^CBD-SPPCD	^‡^MRI: diffuse atrophy; mild hippocampal atrophy, more marked than the rest of the brain	Bilateral temporo-parietal hypoperfusion
9	^||^AD-SPPCD	^‡^MRI: diffuse cortical atrophy	Not done
10	^¶^CBD-SPPCD	^‡^MRI: Diffuse atrophy, especially posterior. Very mild leukoaraiosis.	Bilateral temporo-parietal posterior hypoperfusion with extension to frontal regions
11	^||^AD-SPPCD	^†^CCT: mild right parietal atrophy	Bilateral temporo-parietal hypoperfusion, more evident on right, with mild extension to right frontal region
12	**DLB-SPPCD	^‡^MRI: global severe atrophy, moderate leukoaraiosis	Bilateral temporo-parietal hypoperfusion, more evident on the left

* SPPCD, syndrome of progressive posterior cortical dysfunction;

† CCT, cranial computed tomography,

‡ MRI, magnetic resonance imaging;

§ SPECT, single photon emission computed tomography.

|| AD-SPPCD, patients with Alzheimer's disease meeting criteria for SPPCD;

¶ CBD-SPPCD, patients with corticobasal degeneration meeting criteria for SPPC;

** DLB-SPPCD, patient with dementia with Lewy body meeting criteria for SPPCD.

Ten patients had been submitted to conventional single photon emission computed tomography (SPECT) (Table 2). Bilateral temporo-parietal hypoperfusion, more marked on one side, was observed in five patients. More focal or asymmetric findings were present in the other five patients.

Clinical diagnoses were possible or probable AD in seven patients, CBD in four, and DLB body in one.

## Discussion

We described a group of twelve patients who all presented predominant progressive higher order visual symptoms. When hearing the patient’s or caregiver’s complaints, the clinician should suspect dysfunction of the central visual processing pathways and check for visual acuity and presence of simultanagnosia, optic apraxia, ocular ataxia, visual agnosia and other features of SPPCD, so called “conventional parietal symptoms”, such as limb apraxia and hemineglect.

The presenile onset in all our cases is congruent with the majority of studies to date on SPPCD.^1,3,4,24^ In our case series, there was only one patient with visuoperceptive symptoms. The lower frequency of cases presenting with visuoperceptive symptoms is similar to literature reports.^1,3,4,24^ Patients’ visual difficulties and advanced stages of disease may have accounted for the very low MMSE scores in our sample. Impairment in visual processing might also have contributed to the low recall scores of simple line drawings from the Brief Cognitive Screening Battery. Although loss of memory was not the main complaint, most of the patients presented objective memory impairment in the tests.

Besides the uniformity of the SPPCD, patients frequently presented with additional symptoms and signs at onset or during the course of this syndrome which may be suggestive of several other degenerative diseases.

It is important to comment on the relationship between the diagnosis of CBD and SPPCD. The classic description and diagnostic criteria of CBD^12^ apparently differ significant from SPPCD criteria. However, in everyday practice at a tertiary cognitive out-patient clinic, we occasionally encounter patients with overlapping symptoms of both conditions.

The symptom most strongly linked to CBD is apraxia. The limb apraxia is virtually always asymmetric and is most often ideomotor: patients are typically impaired in using tools, mimicking tool use and imitating mimes of tool use, while recognition of actions is usually relatively preserved.^25^ Visuospatial skills, when assessed, are found to be impaired: poor performance on the Benton’s Judgment of Line Orientation Test^26,27^ and severe deficits in mechanical problem solving, which authors regarded as a predominantly high-level visuospatial ability linked to dorsal stream functions.^28,29^ Because the locus of pathology in CBD involves dorsal occipito-parietal, rather than more ventral occipito-temporal visual pathways, consequently such tasks should reveal selective breakdown in spatial processing. Recently, Mendez^30^ reported a patient with CBD and severe visuospatial impairment, in addition to all the features of Bálint’s syndrome.

In our sample of patients, there are six patients with overlapping symptoms of CBD syndrome and SPPCD. Five of these patients had CBD as their final clinical diagnosis. Asymmetric parkinsonism was present in all of these patients, alien hand phenomena in 3, hemineglect in 3, prominent asymmetric apraxia syndrome in 6 and myoclonus in 2. These patients presented predominantly high-order visual symptoms and clinical criteria of CBD at first or second consultation. Early parkinsonism, especially asymmetric, complaints that suggested very prominent apraxia syndrome, alien hand phenomena and cortical sensory deficits, called our attention. In fact, it is recommended that the cognitive evaluation of patients with suspected CBD should include not only a global dementia measure, such as the MMSE, but also at least a brief assessment of frontal/executive, visuospatial and language function.^26^

Also worthy of comment is the relationship of SPPCD with visual hallucinations and DLB. Visual hallucinations have been reported to occur in up to 25% of patients who met criteria for PCA.^3,31^ Josephs et al.^32^ studied clinical and imaging features of 59 patients with SPPCD and divided them into two groups: with (N=13) and without hallucinations (N=46). Hallucinations were associated with parkinsonism, rapid eye movement sleep behavior disorder and myoclonic jerks. Voxel-based morphometry results suggested hallucinations might involve a circuit of thalamocortical connections. The visual hallucinations were well formed, recurrent and spontaneous. All the patients with visual hallucinations met the clinical research criteria for probable DLB,^13^ although not at the base line evaluation.

Our series of cases included one patient who met clinical criteria for probable DLB around 2 years after SPPCD onset. The visual hallucinations were persistent, well formed and spontaneous and the neuropsychological profile consistent with DLB. These findings suggest that a patient who presents with the SPPCD may later progress into a DLB-like phenotype.

The presence of other features besides typical SPPCD, lead us to consider the differential diagnosis found in clinicopathologic studies.

The findings above reinforce the multiple etiologies of SPPCD. This does not however necessarily imply AD. SPPCD has also been reported in some patients with Pick’s disease^7^ and Creutzfeldt-Jakob disease (Heidenhain’s type).^7,33,34^ The typical presentation of these diseases has features which are very distinct from SPPCD but in some circumstances symptoms may also overlap.

**Figure 2:**
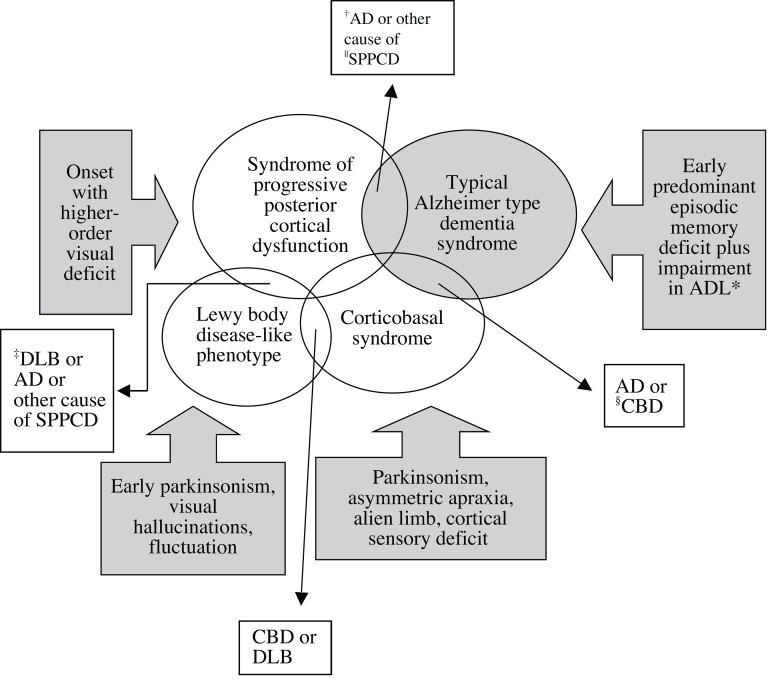
Diagram of overlapping clinical syndromes. *ADL, activities of daily living; ^†^AD, Alzheimer’s disease; ^‡^DLB, dementia with Lewy body; ^§^CBD, corticobasal degeneration; ^||^SPPCD, syndrome of posterior progressive cortical dysfunction.

A diagram of the overlapping syndromes is shown in Figure 2.

In a recent study, forty patients with SPPCD were examined, nine of whom died and underwent autopsy. Brains of seven patients had AD pathology with neurofibrillary tangles (NFT) distributed predominantly in Brodmann’s areas 17 and 18. Two cases showed corticobasal degeneration pathology. Complete and partial Bálint syndrome was found in 35 patients. Five patients had developed visual hallucinations and six parkinsonism during their illnesses. Two of these patients had AD and DLB at autopsy.^3^

Studies on neuropathology and anatomoclinical correlation concerning major damaged pathways in patients with AD, Bálint syndrome and SPPCD have found a global caudal shift in pathology, suggesting that the connections underlying this functional component of the visual system were “devastated”, whereas they are normally less impaired in typical AD.^35-41^ Indeed, these papers suggest that there is not simply a caudal displacement in pathology, but also a selective loss of visual function. These patients were not blind nor did they have problems in form, shape or color recognition. The visual defects specifically affect the analysis of space and the detection of motion.

With regard to the clinical aspect, in 1909, Reszö Bálint originally described a complex visual syndrome which was first documented in an AD patient by Grünthal (cited by Hof et al.).^35^ More recently, Mendez and Cherrier^42^ have suggested an evolution of SPPCD progressing from a difficulty in visual integration beginning with letters, followed by impairment in interpretation of whole scenes, and finally culminating in Bálint’s syndrome.

In contrast to the numerous studies on Bálint syndrome and visuospatial disturbance, less attention has been dedicated to visual agnosia. This may be because the condition is not usually reported, unlike visuospatial deficits. However, there have been a few case reports and series of patients studied clinically and/or pathologically that presented visual object agnosia.^31,35,36,38,41,43-45^ The detailed pathological analysis of autopsies found a more extensive disconnection of a specific component of corticocortical visual associative systems. The distribution of lesions correlated well with neurological symptomatology presented and supports the hypotheses that long corticocortical projections are disrupted in AD.^46^ Another study^41^ found a correlation between the NFT densities in Brodmann’s areas 18, 19 and 37 and associative visual agnosia, whereas NFT in the areas studied did not correlate with apperceptive visual agnosia. These authors suggest that such results support the existence of a dichotomy between associative and apperceptive agnosia, showing that only the former is related to damage of secondary and high-order visual association areas in AD.

We had a patient in our case series whose first manifestation was associative visual agnosia. She was unable to recognize objects but could name accurately them when allowed to touch them.

The findings of structural imaging have always drawn researcher’s attention. In fact, early studies considered the finding of asymmetric posterior atrophy as important as the clinical picture *per se*. As more research groups began to recognize and follow these patients more closely, it became clear that focal posterior asymmetric atrophy is not mandatory for the diagnosis and may appear in late stages of the disease.

In our case series, three patients showed more focal atrophy while all presented diffuse atrophy ranging from mild to severe. All patients with AD showed cortical atrophy, and one patient with CBD presented left focal parietal atrophy. The retrospective nature of the study coupled with the fact that the patients were submitted to CCT or MRI in different stages of the disease might have accounted for these heterogeneous findings.

Previous articles on functional imaging include several single case reports and a few series of small samples.^32,42,43,45,47-50^ The most common finding of functional imaging in SPPCD is hypoperfusion or hypometabolism (with SPECT or positron emission tomography, respectively) in occipital and posterior parietal areas, usually more marked on the right side. Occasionally the temporal associative cortex is involved and frontal extension found.^45,50^ The frontal eye field hypometabolism may result from secondary loss of input from the occipito-parietal region. In our study, ten patients were submitted to conventional SPECT. Unilateral asymmetric findings were present in 3 patients. One of the patients clinically diagnosed with CBD presented left temporo-parietal hypoperfusion. The patient whose associate visual symptom was agnosia, showed marked bilateral temporal hypoperfusion. SPECT findings were also very heterogeneous. Unfortunately, the majority of the patients were submitted to SPECT only at late stages of the disease.

There are several major limitations in our study. It is a retrospective study based on chart information. Patients had been evaluated by several neurologists over the years, who had not been specifically searching for symptoms and signs resulting from posterior cortical impairment. Secondly, there was no neuropathological confirmation of the diagnosis in any of the patients. The absence of neuropathology studies leaves us with a presumptive diagnosis. Also, the patients did not undergo an ophthalmologic consultation, which would have been valuable to accurately measure their visual acuity and thus ensure the visual deficits were entirely or predominantly secondary to central associative visual pathways dysfunction. Another limitation is the profile of patients analyzed, since the group of patients was very heterogeneous with individuals having been examined at various stages of disease. The complete neuropsychological evaluation could not be done in all the patients, especially since some were already at moderate stages of the disease while social problems prevented them from returning regularly to our outpatient clinic.

However, this manuscript demonstrates the difficulties in classifying patients into groups of predetermined criteria, not to mention the frequency with which we encounter patients with overlapping features. Therefore, it is more prudent and didactic to classify patients into syndromes that may overlap at some point during the course of the disease. As the disease progresses, it becomes easier to classify the patients under specific clinical diagnostic criteria.

This paper aimed to raise physicians and researchers’ awareness concerning this rare but important clinical entity. This awareness leads us to a better understanding of SPPCD and its association with other neurodegenerative diseases. Clinicians should consider this diagnosis in relatively young patients who have slowly progressive non-ocular visual complaints.

## APPENDIX

### Illustrative case report

*Case MNA* – A 56-year-old right-handed ex-stewardess presented to the Behavioral and Cognitive Unit with a one-year history of progressive spatial disorientation. It first affected neighborhood disorientation and progressed to home disorientation. She kept repeating the same ideas. The caregiver also complained that the patient could not see what was in front of her and, when reaching for objects (such as door handle or sink tap), she missed them, and relied more on her touch than on eye sight. Family members noticed that she had begun having difficulty in performing home tasks some two years earlier. She needed help in instrumental activities of daily living, for instance, she was unable to use the phone, because she did not see the handset cradle and the numbers, she was unable to wash the dishes as she kept searching for the tap. She was confused with right and left hands. Throughout the course of the disease, the symptoms deteriorated and she had to be aided in basic activities of daily living, presented word-finding difficulties and worsened recent memory. She had discrete dyslipidemia and controlled hypothyroidism. She presented depressed mood. No family history of dementia was reported. On examination, she scored 9 out of 30 points on the MMSE. Mild asymmetric rigid-akinetic parkinsonism was present, being more prominent on the left side. She had difficulty in performing manual tasks under visual guidance bilaterally (optic ataxia), jerky intrusions when attempting to perform smooth pursuit eye movements (ocular apraxia) and could not see two objects at the same time (for instance, a pen and a watch) (simultanagnosia). When the examiner showed some dark line drawings pointing at one for her to name, she could only see the examiner’s fingernail. She had difficulties in performing commands that required right and left orientation. There were normal optic fundi, brisk symmetric tendon reflexes and no frontal releasing signs. On subsequent examinations, the visual signs, recent memory and language functions, especially naming, reading and writing, deteriorated. She presented bilateral finger agnosia, total left spatial and somatosensory extinction, motor right hand extinction, intense bilateral ideomotor apraxia and acalculia. Insight and social adequacy were preserved until moderate stages of the disease. She presented a severe difficulty on confrontation field examination, because most of the time she could not see the target (simultanagnosia, ocular apraxia).

Neuropsychological evaluation evidenced intense visuospatial and visual analytic deficits. She could not perform the Hooper Visual Organization Test or the colored Progressive Raven Matrices. She was also unable to recognize the components of the Stanford-Binet Thematic Figure. The visual impairment precluded the evaluation of the other cognitive domains, especially language functions. She scored 69 out of 144 points in the Mattis dementia rating scale, with impairments in all domains, but particularly memory and constructional abilities. Severe difficulty was also seen in the Block Design Test, Trail Making A and Stroop tests. The MRI showed generalized atrophy with prominent bilateral parieto-occipital atrophy. The SPECT showed prominent hypoperfusion of parietal regions, especially on the right side and with right fronto-temporal extension. She was treated with cholinesterase inhibitors (rivastigmine and donepezil) and responded very poorly.
